# Objective and subjective stress, personality, and allostatic load

**DOI:** 10.1002/brb3.1386

**Published:** 2019-08-25

**Authors:** Dinne S. Christensen, Nadya Dich, Trine Flensborg‐Madsen, Ellen Garde, Åse M. Hansen, Erik L. Mortensen

**Affiliations:** ^1^ Section of Environmental Health Department of Public Health University of Copenhagen Copenhagen Denmark; ^2^ Center for Healthy Aging University of Copenhagen Copenhagen Denmark; ^3^ Section of Social Medicine Department of Public Health University of Copenhagen Copenhagen Denmark; ^4^ Danish Research Centre for Magnetic Resonance Centre for Functional and Diagnostic Imaging and Research Copenhagen University Hospital Hvidovre Denmark; ^5^ National Research Centre for the Working Environment Copenhagen Denmark

**Keywords:** allostatic load, Big Five personality traits, major life events, perceived stress, stress

## Abstract

**Introduction:**

Despite the understanding of allostatic load (AL) as a consequence of ongoing adaptation to stress, studies of the stress–AL association generally focus on a narrow conceptualization of stress and have thus far overlooked potential confounding by personality. The present study examined the cross‐sectional association of objective and subjective stress with AL, controlling for Big Five personality traits.

**Methods:**

Participants comprised 5,512 members of the Copenhagen Aging and Midlife Biobank aged 49–63 years (69% men). AL was measured as a summary index of 14 biomarkers of the inflammatory, cardiovascular, and metabolic system. Objective stress was assessed as self‐reported major life events in adult life. Subjective stress was assessed as perceived stress within the past four weeks.

**Results:**

Both stress measures were positively associated with AL, with a slightly stronger association for objective stress. Adjusting for personality traits did not significantly change these associations.

**Conclusions:**

The results suggest measures of objective and subjective stress to have independent predictive validity in the context of personality. Further, it is discussed how different operationalizations of stress and AL may account for some of the differences in observed stress–AL associations.

## 1 INTRODUCTION

Chronic stress exposure has consistently been linked to adverse health outcomes (Cohen, Janicki‐Deverts, & Miller, 2007; Lantz, House, Mero, & Williams, 2005; Renzaho et al., 2014). Over the past decades, an increasing number of studies have suggested that this association arises through physiological dysregulation, or allostatic load (AL) (Beckie, 2012; McEwen & Stellar, 1993). Specifically, it is posited that the physiological response facilitating short‐term adaptation to stress can become maladaptive if repeatedly activated by chronic stress exposure. Over time, this repeated activation will lead to a progressive physiological dysregulation, ultimately resulting in disease and premature mortality. The AL model represents a framework to study the mechanisms underlying the link between stress and disease, as well as a method of quantifying the physiological toll associated with chronic stress. Though there is still no consensus on the optimal method of AL index construction, different AL measures have been shown to predict increased risk of morbidity, decline in physical and cognitive functions, and premature mortality (Beckie, 2012). The empirical support for an association of stress with AL is limited, but growing (Beckie, 2012; Juster, McEwen, & Lupien, 2010). However, as in the literature on stress and disease more generally (Epel et al., 2018), the field is characterized by diversity in the definition and measurement of stress. One area of divergence concerns the emphasis placed on objective versus subjective aspects of stress. The aims of the present study were (a) to test and compare the associations of two different measures of stress with AL and (b) to examine the possible confounding of these associations by Big Five personality traits.

According to the stimulus‐oriented theory of stress, stress can be characterized as external events (stressors) that elicit a change or increase in demands to the individual, necessitating adaptation (Derogatis & Coons, 1993). Within this conceptualization, stress is typically measured using a checklist of major life events (MLE) believed to be objectively stressful (Holmes & Rahe, 1967). In contrast, the transactional theory of stress emphasizes the individual's *perception* of stress as central to the impact of a given stressor (Lazarus & Folkman, 1984). This approach questions the assumption that certain events are inherently stressful, focusing on the subjective perception of, rather than objective exposure to, stress. In this field, a well‐established measure of stress is self‐reported perceived stress, for example assessed by the Perceived Stress Scale (PSS, Cohen, Kamarck, & Mermelstein, 1983). Within the stress literature, it has been indicated that objective and subjective measures of stress in fact measure different things, and may have different pathogenic consequences (Cohen, Tyrrell, & Smith, 1993). Still, despite the central role of stress in the AL framework, most studies on the stress–AL association have focused on only one of these constructs. For example, it has been argued that AL represents a mechanism by which exposure to objective stress “gets under the skin,” though evidence is mixed (Evans, 2003; Mair, Cutchin, & Kristen Peek, 2011; Schulz et al., 2012; Turner, Thomas, & Brown, 2016). Subjective stress has also been studied as a possible determinant of AL, with findings predominantly (though not exclusively, see [Graves & Nowakowski, 2017]) suggesting weak but significant positive associations, in some studies indicated to be stronger in women compared to men (Glei et al., 2013; Goldman, Glei, Seplaki, Liu, & Weinstein, 2005; Upchurch et al., 2015).

However, a few studies have included both understandings of stress. One study compared the effects of environmental (objective) stress on AL with that of psychological (subjective) stress in dementia caregivers (Clark, Bond, & Hecker, 2007). Environmental stress was operationalized as a weighted index of stressful life events in the past three years, while psychological stress was assessed using the PSS. Cross‐sectionally, stressful life events were significantly associated with secondary outcomes (cardiovascular and metabolic markers) of the AL index, while prospectively, a stronger association was found of perceived stress with primary mediators (neuroendocrine markers). Overall, perceived stress was concluded to be a stronger predictor of AL, and it was recommended that studies on AL as an outcome of stress should include a distinction between primary mediators and secondary outcomes, and between objective and subjective measures of stress (Clark et al., 2007). Another study examined associations of AL with negative life events using a 51‐item version of the revised social readjustment rating scale, chronic stress measured as self‐reported presence of stress in 8 life domains (e.g., financial, marital), and perceived stress assessed using the PSS (Hawkley, Lavelle, Berntson, & Cacioppo, 2011). Only perceived stress was significantly associated with AL. Finally, one study examined whether the association of a number of stressors (life events) with AL was mediated by perceived stress measured as self‐reported level of stress in different life domains (Glei, Goldman, Chuang, & Weinstein, 2007). Both number of stressors and perceived stress were positively associated with AL, with a slightly stronger association for perceived stress and limited evidence for mediation. Overall, studies including both subjective and objective measures of stress appear to suggest stronger associations of subjective stress with AL.

These results are surprising considering the conceptualization of AL as reflecting the physiological consequences of cumulative stress (Seeman, Singer, Rowe, Horwitz, & McEwen, 1997). It seems counterintuitive that measures of subjective stress, usually reflecting levels of perceived stress in a recent and limited time period, should be stronger predictors than objective stress measured as major life events over the course of several years. However, most previous studies have disregarded a potential source of error which may partly explain these findings: the influence of personality. Already in the original formulation of the AL framework, it was described how stable, individual differences in the propensity to interpret stimuli as stressful was expected to contribute to AL levels (Seeman et al., 1997). Subjective stress measured as perceived stress is a state‐measure, and as such is expected to fluctuate over time, in contrast to personality measures, which are distinguished in part by their stability over time (Matthews, Deary, & Whiteman, 2009). Specifically when studying an outcome such as AL, one would expect the predictive validity of trait measures to be superior to that of state‐measures. Further, measures of perceived stress by definition emphasize the subjective appraisal of objective circumstances (Cohen, Kessler, & Gordon, 1997). As such, these measures will be influenced by a range of factors related to the individual, for example psychological symptoms, concurrent mood states, and personality dispositions (Ebstrup, Eplov, Pisinger, & Jørgensen, 2011; Roohafza et al., 2016). In fact, neuroticism can be characterized as “an enduring disposition to experience psychological distress” (Costa & McCrae, 1990, p. 23), and measures of perceived stress and neuroticism tend to have items that overlap or resemble each other closely, such as “I often feel tense and jittery” (Costa & McCrae, 1989). This introduces common method variance, compromising the discriminant validity of perceived stress measures (Podsakoff, MacKenzie, Lee, & Podsakoff, 2003). The remaining traits are all inversely associated with PSS scores (Ebstrup et al., 2011), and high levels of extraversion, openness, and conscientiousness are associated with less stressor‐related negative affect (Leger, Charles, Turiano, & Almeida, 2016). Following the standard definition of confounding, a confounder is a factor that is associated with the outcome and the exposure, which is not an intermediate variable in the pathway between them, and, when not controlled for, distorts the estimate of their association (Szklo & Nieto, 2014). Because personality is also associated with AL (Christensen, Flensborg‐Madsen, Garde, Hansen, & Mortensen, 2019; Stephan, Sutin, Luchetti, & Terracciano, 2015), it can thus be considered a potential confounder to the extent that it, if disregarded, distorts the estimate of the influence of perceived stress. While this does not mean that the association of perceived stress with AL is necessarily spurious, it should be interpreted with attention to personality in order to disentangle the effect associated specifically with perceived stress levels (Costa & McCrae, 1990).

While measures of objective stress based on life events are neither state nor trait measures, they generally reflect stress exposure over a prolonged period (Cohen et al., 1997) and should ideally be less influenced by the mood and immediate conditions of the respondent. However, an influence of personality cannot be excluded. Through processes known as situational selection or “niche picking” (Snyder, 1983), individuals tend to seek out environments and experiences that are congruent with their personality. For example, extraversion has been found to prospectively predict positive events and neuroticism adverse events, whereas openness predicts both types of life events (Headey & Wearing, 1989; Kendler, Gardner, & Prescott, 2003; Lüdtke, Roberts, Trautwein, & Nagy, 2011; Magnus, Diener, Fujita, & Pavot, 1993; Sutin, Costa, Wethington, & Eaton, 2010). While empirical findings regarding agreeableness and conscientiousness are more sparse (Hampson et al., 2016; Lüdtke et al., 2011; Vaidya, Gray, Haig, & Watson, 2002), both could theoretically be expected to be inversely associated with objective stress (agreeableness through positive social relations and conflict avoidance; conscientiousness through a well‐structured, healthy, and productive lifestyle). It has previously been shown that personality indirectly predicts health problems and physiological dysregulation through stressful life events (Hampson et al., 2016; Iacovino, Bogdan, & Oltmanns, 2016), making personality relevant in studies of AL and objective stress as well.

Finally, personality could moderate the associations of both stress measures with AL in several ways (Hampson, 2012). Both the affective and biological stress responses have been found to vary depending on personality (Childs, White, & de Wit, 2014; Oswald et al., 2006), and systematic associations have been found among personality traits and coping styles (Carver & Connor‐Smith, 2010). For AL specifically, psychosocial vulnerability has been shown to moderate the association of objective stress with AL (Glei et al., 2007).

To our knowledge, the present study is the first to examine and compare the associations of objective and subjective measures of stress with AL in the context of Big Five personality traits. Based on previous findings and theoretical considerations, both measures of stress are hypothesized to be positively associated with AL. Further, we hypothesize that the association of subjective stress with AL will be attenuated by adjustment for personality to a larger extent than objective stress.

## 2 METHODS

### 2.1 Participants

Data for the present study come from the Copenhagen Aging and Midlife Biobank (CAMB). Established in 2009 with the purpose of studying the aging process across the life course, CAMB is a merger of three existing cohorts: The Metropolit Cohort consisting of men born in Copenhagen in 1953 (Osler, Lund, Kriegbaum, Christensen, & Andersen, 2006), the Copenhagen Perinatal Cohort consisting of men and women born at the National University Hospital in Copenhagen in 1959–1963 (Zachau‐Christiansen & Ross, 1975), and the Danish Longitudinal Study on Work, Unemployment, and Health consisting of a random sample of Danish men and women born in 1949 and 1959 (Christensen et al., 2004). A total of 17,937 cohort members residing in the eastern parts of Denmark were invited. Of these, 7,189 (40%) participants answered a postal questionnaire and 5,575 (31%) participants underwent a health examination conducted at the National Research Centre for the Working Environment from 2009 to 2011. A detailed description of the recruitment procedure, including measures and data collection, is available elsewhere (Lund et al., 2016). The local ethics committee has approved the CAMB as a database combining three cohorts (No: H‐A‐2008‐126), and CAMB has been registered at the Danish Data Protection Agency as a combined database (No: 2008‐41‐2938). All participants have provided informed consent.

### 2.2 Measures

#### 2.2.1 Allostatic load

Nonfasting blood samples were collected and immediately analyzed for cholesterol, glucose, and hemoglobin. Blood samples were frozen and stored at −20°C and analyzed for HbA1c, lipids, and levels of several inflammatory markers within 12 months. A more detailed description can be found in Avlund et al. (2014). As part of the health examination, systolic and diastolic blood pressure was measured two times on each arm, and participants' height, weight, and body fat percentage were measured (Hansen et al., 2014). Allostatic load was calculated as a simple summary index using the traditional method from Seeman et al. (1997) of summing the number of biomarkers falling in the sex‐specific, high‐risk sample quartile. The present study included three inflammatory markers representing primary mediators and 11 cardiovascular and metabolic markers representing secondary outcomes. Table 1 shows the included biomarkers with sex‐specific means and cut‐points. With a total of 14 biomarkers, the theoretical index range was 0–14, with higher scores indicating higher AL. Information on one or more biomarkers was missing for 3% of the study sample. Both the full AL score and each subsystem score were calculated only for participants with information on at least half of the included biomarkers. In the case of less than half of the biomarkers missing, full AL or subsystem score was calculated as the mean score of the available biomarkers multiplied by the full number of biomarkers. Only participants with sufficient biomarkers to compute the full AL score were included, resulting in the exclusion of 63 participants for whom 8 or more biomarkers were missing. The final sample thus consisted of 5,512 participants with AL scores.

**Table 1:** Biomarkers and cut‐points. Sex‐stratified means and sample‐defined risk cut‐points for 5,575 participants with blood samples

	Men				Women			
	*N*	Mean	*SD*	Cut‐point	*N*	Mean	*SD*	Cut‐point
Primary mediators								
hsCRP (mg/ml)	3,760	2.34	4.33	>2.40	1,721	2.29	4.33	>2.30
Interleukin‐6 (pg/ml)	3,761	3.73	14.4	>3.04	1,722	3.40	16.8	>2.65
TNF alpha (pg/ml)	3,764	6.11	12.9	>5.76	1,722	5.54	14.7	>5.34
Secondary outcomes								
Diastolic blood pressure (mmHg)	3,818	89.5	10.4	>96	1,750	84.6	10.4	>90.8
Systolic blood pressure (mmHg)	3,818	139.3	16.6	>149	1,750	126.2	16.7	>135.5
HbA1c (% of total hemoglobin)	3,779	5.43	0.59	>5.68	1,729	5.21	0.49	>5.47
Total cholesterol (mmol/l)	3,774	6.18	1.16	>6.90	1,730	6.19	1.10	>6.88
High‐density lipoprotein (mmol/l)	3,774	1.39	0.36	<1.14	1,730	1.69	0.42	<1.41
Low‐density lipoprotein (mmol/l)	3,774	3.05	0.85	>3.59	1,730	2.99	0.87	>3.50
Triglycerides (mmol/l)	3,773	1.95	1.17	>2.33	1,729	1.45	0.74	>1.75
Body mass index (kg/m^2^)	3,816	26.5	3.89	>28.5	1,753	25.3	4.77	>27.5
Waist/Hip ratio	3,804	0.95	0.06	>0.99	1,750	0.85	0.06	>0.90
Body fat (%)	3,794	21.4	5.81	>24.9	1,747	31.3	6.63	>35.8
Blood glucose (mmol/l)	3,796	5.65	1.65	>6.10	1,741	5.32	1.10	>5.70

Abbreviations: HbA1c, Glycated hemoglobin; hsCRP, High sensitivity C‐reactive protein; TNF alpha, Tumor necrosis factor alpha.

#### 2.2.2 Objective stress

Objective stress was operationalized as the experience of major life events (MLE). Information on MLE was obtained using a modified short version of the Social Readjustment Rating Scale (Holmes & Rahe, 1967). This version has previously been used to assess the relationship of stressful life events with leukocyte telomere length (Osler, Bendix, Rask, & Rod, 2016) and ischemic heart disease (Andersen, Diderichsen, Kornerup, Prescott, & Rod, 2011). The measure consisted of 11 yes/no self‐report items representing adult private (six items) and work life (five items) events after the age of 20 years.

- MLE in adult private life: Long‐standing or serious school problems of children, prolonged or serious illness of children, death of a child, prolonged or serious illness or death of an adult relative, prolonged or severe marital problems, prolonged or serious financial problems.
- MLE in adult work life: Loss of job, prospect of promotion that never happened, long‐standing or serious conflicts with colleagues, long‐standing or serious conflicts with superiors, long‐standing or serious conflicts with subordinates.

The original scale included an item about prolonged or serious personal illness, which was excluded as it might represent a pathway from objective stress or other covariates to AL, or a consequence of high AL. Further, participants were asked to indicate whether they had ever had a job. For those who indicated that they had never had a job in their adult life (*n* = 41), all work life items were treated as missing. A simple summary index was computed to reflect the number of major life events experienced. The missing data rate was <2% for each item. Participants with less than six of the included items available were treated as missing (*n* = 64). For participants with more than six but less than all 11 items available, the index score was computed as the mean of the available items multiplied by 11.

#### 2.2.3 Subjective stress

Subjective stress was operationalized as self‐reported perceived stress within the past four weeks, assessed by four items from the short version of the Copenhagen Psychosocial Questionnaire (Pejtersen, Kristensen, Borg, & Bjorner, 2010). Items in this scale were specifically selected to avoid overlap with environmental stress measures. Participants were asked how often within the past four weeks they had experienced any of the following: problems relaxing, irritableness, tension, stress. Original responses ranged from 1 “All of the time” to 5 “At no time.” These were reverse‐coded and recoded for a range of 0–4 for each item, so that the final simple summary score ranged from 0 to 16 with higher scores reflecting higher levels of perceived stress. Cronbach's alpha for the four items was .81. The missing data rate was below 2% for all items. Participants with less than two available items were treated as missing (*n* = 72). For participants with at least two but less than all four items available, the score was computed as the mean of the available items multiplied by four.

#### 2.2.4 Personality

Personality was assessed with the NEO Five‐Factor Inventory (NEO‐FFI), the short version of the Revised NEO Personality Inventory (NEO PI‐R) (Costa & McCrae, 1989). Details regarding the construction of the Danish version of the NEO‐FFI are described in Mortensen et al. (2014). Based on 60 items in 0–4 Likert formats (strongly disagree to strongly agree), the NEO‐FFI assesses trait neuroticism, extraversion, openness, agreeableness, and conscientiousness. Each trait is based on 12 items for a theoretical score range of 0–48. In the present sample, Cronbach's alphas for each trait were as follows: neuroticism .85, extraversion .81, openness .74, agreeableness .69, and conscientiousness .79.

#### 2.2.5 Covariates

A set of covariates were included to account for potential confounding and for natural variation in biomarker levels. These include sex, age, time of blood draw, fasting status within two hours before blood draw, and years of education. Years of education included school education and vocational training. School education ranged from 8 years (completion of primary and/or secondary schooling without passing an examination) to 12 years (high school examination). Vocational training ranged from 0 years (no training) to five years (long university education). The combined education score thus ranged from 8 to 17.

### 2.3 Statistical analyses

Sex‐stratified means or percentages for all study variables were calculated. In preliminary analyses, sex differences were tested using *t* tests or chi‐square tests and zero‐order correlations among AL, stress measures, and personality traits were examined. The stress–AL association was tested in a series of hierarchical regression models. First, AL was regressed on objective stress while adjusting for covariates (Model 1). Next, subjective stress was added to compare the relative strength of the two measures (Model 2). Potential interaction effects of sex with each stress measure were tested in models containing only the stress measure and covariates. Finally, all Big Five personality traits were included in one step due to trait intercorrelations (Model 3). Standardized coefficients are reported. All models used robust standard error estimation methods and were based on full information maximum likelihood (FIML), allowing for the inclusion of incomplete data under the missing at random assumption. The missing data rate ranged from 0% (AL, sex, age) to 1.5% (years of education).

Additionally, two supplementary analyses were performed. First, to test whether personality traits modify the stress–AL associations, two‐way interaction terms of each personality trait with objective and subjective stress were tested in models including only the stress measure of interest, remaining personality traits, and covariates. Second, model 3 was additionally estimated using AL primary mediators and secondary outcomes as dependent variable in order to examine potential differences in the biological mechanisms of the two stress measures.

Finally, two sensitivity analyses were conducted. First, because recent infections may influence biomarker levels, all main regression models were adjusted for self‐reported systemic infections within the past 3 weeks (fever, cold, flu, pneumonia, digestive or urinary tract infection, or other infections). Second, potential bias due to missing data was assessed in a sensitivity analysis using complete cases only. All analyses were conducted in Stata V14.

## 3 RESULTS

Means and standard deviations for all variables and *p*‐values for tests of sex differences are presented in Table 2. The sample consisted of 69% (3,782) men. There were no significant sex differences in AL. Compared to men, women reported more life events and higher levels of perceived stress.

**Table 2:** Descriptive statistics of allostatic load, stress measures, personality, and covariates

Variables	Men		Women		*p* a
	*n*	Means (*SD*; range) or *N* (%)	*n*	Means (*SD*; range) or *N* (%)	
Allostatic load	3,782	3.44 (2.53; 0–12)	1,730	3.44 (2.66; 0–13)	.96
Primary mediators	3,764	0.75 (0.91, 0–3)	1,722	0.74 (0.93; 0–3)	.94
Secondary outcomes	3,782	2.70 (2.14; 0–10)	1,730	2.70 (2.17; 0–10)	.93
Objective stress (major life events)	3,733	2.43 (1.71; 0–9)	1,715	2.54 (1.75; 0–9)	.023
Subjective stress (perceived stress)	3,727	3.58 (2.52; 0–16)	1,713	3.96 (2.70; 0–15)	<.001
Neuroticism	3,748	16.9 (6.98; 0–46)	1,716	19.3 (7.18; 2–45)	<.001
Extraversion	3,760	30.7 (6.21; 2–48)	1,715	31.1 (6.28; 10–47)	.020
Openness	3,748	28.1 (6.23; 6–46)	1,715	28.9 (6.08; 12–45)	<.001
Agreeableness	3,748	32.7 (5.21; 11–48)	1,717	35.1 (4.86; 16–47)	<.001
Conscientiousness	3,748	33.5 (5.55; 12–48)	1,716	33.7 (5.39; 6–47)	.38
Age	3,782	55.3 (3.32; 49–63)	1,730	52.5 (4.37; 49–63)	<.001
Time of blood draw (AM)	3,781	11.1 (2.40; 7–17)	1,729	11.4 (2.30; 7–17)	<.001
Fasting status, fasting	3,782	2,324 (61.5)	1,730	1,111 (64.2)	.14
Years of education	3,717	13.1 (2.58; 8–17)	1,711	13.4 (2.20; 8–17)	<.001

a *t* test or chi‐square tests for sex differences.

The zero‐order correlations of all variables displayed in Table 3 showed weak, but positive associations of both stress measures with AL: *r* = .07 for objective stress and *r* = .06 for subjective stress (both *p* < .001). For both stress measures, the strongest trait correlation was found for neuroticism, so that higher levels of neuroticism were associated with higher levels of both objective (*r* = .21, *p* < .001) and subjective stress (*r* = .50, *p* < .001). The correlation of subjective stress with neuroticism was, however, more than twice that of objective stress. Extraversion and conscientiousness also correlated most strongly with subjective stress (*r *= −.17, *p* < .001 and *r *= −.24, *p* < .001, respectively), whereas agreeableness showed parallel correlations for objective and subjective stress (both *r* = −.12, *p* < .001). Openness was significantly associated with objective stress only (*r* = .15, *p* < .001).

**Table 3:** Correlation matrix of study variables

	1	2	3	4	5	6	7	8	9
1. Allostatic load	–								
2. Primary mediators	0.60***	–							
3. Secondary outcomes	0.94***	0.29***	–						
4. Objective stress	0.07***	0.05***	0.07***	–					
5. Subjective stress	0.06***	0.04**	0.05***	0.21***	–				
6. Neuroticism	0.06***	0.05***	0.05***	0.21***	0.50***	–			
7. Extraversion	−0.05***	−0.04**	−0.04**	−0.03*	−0.17***	−0.44***	–		
8. Openness	−0.10***	−0.07***	−0.08***	0.15***	0.001	−0.02	0.35***	–	
9. Agreeableness	−0.01	−0.002	−0.01	−0.12***	−0.12***	−0.12***	0.01	0.01	–
10. Conscientiousness	−0.10***	−0.08***	−0.08***	−0.12***	−0.24***	−0.54***	0.35***	0.07***	0.13***

* *p* < .05,

** *p* < .01,

*** *p* < .001.

Table 4 presents the results of the multiple regression models regressing AL on objective and subjective stress, adjusting for personality. Adjusted only for covariates, objective stress was weakly but highly significantly associated with AL (β = .082, *p* < .001). Including subjective stress in model 2 only slightly attenuated this association (β = .075, *p* < .001), and the association of subjective stress with AL (β = .036, *p* = .011) was considerably weaker than that of objective stress. There were no significant sex × stress interactions.

**Table 4:** Allostatic load modeled by objective and subjective stress, adjusted for personality traits

	Model 1		Model 2		Model 3	
	β	*p*	β	*p*	β	*p*
Objective stress	.082	<.001	.075	<.001	.078	<.001
Subjective stress			.036	.011	.035	.031
Neuroticism					−.029	.14
Extraversion					.017	.31
Openness					−.047	.003
Agreeableness					−.006	.660
Conscientiousness					−.069	<.001
Sex	.040	.005	.038	.008	.047	.002
Age	.091	<.001	.093	<.001	.096	<.001
Time of blood draw (AM)	.004	.76	.004	.76	.002	.87
Fasting status, fasting	−.040	.003	−.040	.003	−.040	.003
Years of education	−.21	<.001	−.21	<.001	−.18	<.001

Note

Standardized coefficients are reported.

Both stress measures remained significantly associated with AL when adjusted for Big Five personality traits in model 3, though the association of subjective stress with AL was slightly attenuated, mainly in significance level (β = .035, *p* = .031), whereas the association of objective stress was slightly strengthened compared to model 2 (β = .078, *p* < .001). Of all Big Five traits, only openness and conscientiousness were significantly associated with AL in model 3, with higher trait levels associated with lower AL scores.

Supplementary analyses showed no significant stress × personality interactions. Regressing model 3 on the two AL subsystems, both objective and subjective stress were found to be positively associated with primary mediators (β = .052, *p* < .001 and β = .031, *p* = .062, respectively) and secondary outcomes of the AL index (β = .072, *p* < .001 and β = .028, *p* = .082, respectively). However, these associations were not statistically significant for subjective stress. Full results from the subsystem analyses are available in Tables S1 and S2.

Sensitivity analyses adjusting for recent infections attenuated the association of objective stress with AL to a minimal extent, whereas the association of subjective stress with AL was significantly attenuated in model 3 (β = .030 *p* = .068). Results from complete case analyses were highly similar to those based on FIML estimation. These results are available from the first author (DSC) by demand.

## 4 DISCUSSION

The present study examined the associations of objective and subjective stress with midlife AL in the context of Big Five personality traits. In accordance with our hypotheses, weak but statistically significant positive associations with AL were found for both measures of stress. Additionally, both measures of stress remained significantly associated with AL when controlling for the other, supporting their status as conceptually distinct measures with independent predictive power. However, comparing the two measures, the association of objective stress with AL was found to be stronger than that of subjective stress. Though this was anticipated considering the notion of AL as a reflection of chronic stress exposure, it is in contrast to previous studies indicating stronger associations of subjective stress with AL (Clark et al., 2007; Glei et al., 2007; Hawkley et al., 2011).

The finding that both measures of stress are significantly associated with AL in the present study is perhaps explained by the substantially larger sample compared to previous studies, allowing even small effects to reach statistical significance. This, however, does not explain why objective stress was found to be a stronger predictor than subjective stress. A possible explanation for this divergence from previous findings is differences in AL operationalizations. The previous studies all included at least three primary mediators of the neuroendocrine system (norepinephrine, epinephrine, cortisol) and comparatively fewer secondary outcomes (e.g., blood pressure, cholesterol) in the AL index than the present study. Here, primary mediators were represented by inflammatory markers of the immune system (high sensitivity C‐reactive protein, interleukin‐6, and tumor necrosis factor alpha). Neuroendocrine system hormones are characterized by their acute variability in response to stress, and at any given time, neuroendocrine marker levels will reflect both an acute reaction to the immediate environment, and a basal level which, within the AL framework, is hypothesized to become dysregulated over time (McEwen, 2004). It has thus been argued that these markers contain an element of “noise,” which more accurately reflects transient states than consequences of chronic stress (Gersten, 2008, p. 517). It is thus plausible that the AL operationalizations of previous studies are more sensitive to acute perceptions of stress, and less so to events which may have occurred several years ago, whereas the present operationalization may underestimate the effects of subjective (acute) stress. This interpretation is supported by two previous studies showing subjective stress to correlate more strongly with neuroendocrine markers of the AL index than objective stress (Clark et al., 2007; Gersten, 2008). In the supplementary analyses of the present study, objective stress was the strongest predictor of both subsystems, though subjective stress did show stronger correlation with primary mediators compared to secondary outcomes. These findings thus highlight the relevance of the specific construction of the AL index, differences in which have previously been argued to be a weakness for the field of studies based on the AL framework (Johnson, Cavallaro, & Leon, 2017). Specific recommendations regarding the optimal construction are outside the scope of this study, but it is expected to be beyond discussion that the included biomarkers should reflect what AL is posited to measure, namely the consequence of chronic or cumulative stress exposure, rather than transient states.

The contrast in results compared to previous findings may also arise from different operationalizations of stress. Importantly, the present study partly replicates previous findings of a significant subjective stress–AL association, but deviates with respect to the association of objective stress. This may reflect a strength of the subjective measure, that is the relative consistency with which it is operationalized across studies (perceived stress within the past days or weeks). Operationalizations of objective stress are more diverse. Even within measures based on life event checklists, the number, nature, and time span of the included events can vary. For example, whereas the present study included events from the past 30 years, one of the previous studies measured objective stress as events occurring within the past three years (Clark et al., 2007), and in another, only some events were registered as “ever experienced” while others reflected current problems (Glei et al., 2007). Further, an inherent problem with life event measures is that even the same events (recorded over the same time span) can have vastly different expressions across populations. For someone young, the loss of a job may be a short‐lived problem of minor consequence, whereas to someone older it may represent a complete shift in existence (Cohen et al., 1997). This variability alone will reduce the chance of replicating findings across studies. Unfortunately, it seems the only way of overcoming this problem is to include questions pertaining to the appraisal of events, making objective measures what they were designed not to be—subjective (Costa. & McCrae, 1990).

Support for our hypothesis that the association of subjective stress with AL would be more attenuated by controlling for personality than objective stress was very limited. Estimates for both associations were virtually unchanged, and the association of subjective stress remained significant, though slightly less so. This was despite significant bivariate correlations of all personality traits with AL and both stress measures (except agreeableness, which was not significantly associated with AL, and openness, which was not significantly associated with subjective stress), supporting the rationale for their inclusion. This indicates that the stress–AL associations found in previous studies are nonspurious, and validates subjective stress especially as conceptually distinct from personality in general and neuroticism in particular—at least in relation to AL.

### 4.1 Strengths and limitations

Strengths of the present study include the large population‐based sample and the use of an objectively measured index of physiological dysregulation (AL). Compared to specific clinical outcomes such as cardiovascular disease or mortality, the complex multi‐system nature of the AL measure has been argued to be more characteristic for the physiological consequences of stress (Seplaki, Goldman, Weinstein, & Lin, 2004). Additionally, using an objectively measured health outcome rather than measures based on self‐report eliminates bias due to common method variance (Podsakoff et al., 2003). However, several limitations should be mentioned.

The optimal method of stress measurement has been the topic of an ongoing discussion in psychology for decades (see, e.g., Cohen et al., 1997; Costa Jr. & McCrae, 1990; Dohrenwend, Dohrenwend, Dodson, & Shrout, 1984; Lazarus, 1990). While the inclusion of two measures of stress is a strength in the present study, measures of stressor duration, appraisal, coping style, and social support would have been relevant for improved insight to the complete stress process. Further, labeling the two measures as subjective and objective, respectively, is of course a useful but to some extent artificial simplification. Admittedly, the term “objective stress” carries some inherent ambiguity, and it can be argued that no measure of stress is (or even should be) free from subjectivity (Lazarus, 1990). Objective stress was measured as self‐reported major life events using a modified version of the Social Readjustment Rating Scale. Though this type of measure is frequently used in stress research, it does have limitations; it is not by any means an exhaustive measure of stress exposure, and because it is a self‐report, retrospective measure, it is vulnerable to recall bias (Denkova, Dolcos, & Dolcos, 2012). However, the present measure reflects the occurrence of predefined, externally verifiable events, arguably reducing such bias (Costa & McCrae, 1990). Subjective stress was measured as self‐reported perceived stress within the past four weeks. Though the specific measure has previously found limited use, it resembles the well‐validated PSS (Cohen et al., 1983) in its construction (measuring self‐reported perceived stress within the past four weeks) and wording (questions regarding the experience of, for example, stress and irritation). However, it does represent a more limited measure of stress than the PSS.

While the present study discussed the extent to which neuroendocrine markers reflect chronic stress exposure, we do concur that measures of AL should optimally include biomarkers from all regulatory systems posited to be involved in the stress response (neuroendocrine, immune, cardiovascular, and metabolic). As discussed, the lack of neuroendocrine markers in the present operationalization is a weakness, which may have affected the findings, and certainly limits comparability to other studies.

Finally, the cross‐sectional nature of the data means that causality cannot be established. That is, while we assume that the association of objective and subjective stress with AL observed in the present study reflects the physiological consequences of stress exposure, such directionality cannot be inferred. Similarly, the relationship among stress, AL, and personality is likely to be more complex and interactive than cross‐sectional data can reflect. For example, personality has been found to be influenced by major life events (Löckenhoff, Terracciano, Patriciu, Eaton, & Costa, 2009; Specht, Egloff, & Schmukle, 2011), and AL has been found to predict change in extraversion, conscientiousness, and agreeableness (Stephan et al., 2015). Accounting for more complex dynamics would require future research based on longitudinal data. However, the present measure of objective stress was restricted to events from adulthood in an attempt to minimize the influence of events on personality, in which case personality should be conceptualized as a mediator. Considering the evidence for life‐course stability of personality traits (Costa & McCrae, 1994; Roberts, Caspi, & Moffitt, 2001), and findings indicating that (at least for neuroticism) the influence of traits on experiences is stronger than that of experiences on traits (Jeronimus, Riese, Sanderman, & Ormel, 2014), the notion of stress as influenced by personality rather than vice versa seems warranted. Similarly, we included years of education as a potential confounder though it cannot be excluded that it is in fact a mechanism linking objective stress to AL. However, this risk was minimized by excluding life events before the age of 20 years.

## 5 CONCLUSION AND PERSPECTIVES

Both subjective and objective stresses were significantly associated with AL, and both measures remained significant when controlling for the other and for Big Five personality traits. However, objective stress was found to be more strongly associated with AL. These findings contribute to the study of stress and AL in several ways. First, two different measures of stress rarely used in combination are shown to have independent predictive validity. Considering the fact that stress is such a central concept in the AL framework, future research might benefit from including different measures of stress; these results suggest that including only one type does not tell the full story. Further, it would be interesting for future studies to test whether these stress measures differ in the mechanisms by which they become associated with AL. For example, certain behavioral patterns, which increase or decrease AL levels, might be differentially associated with different measures of stress. Second, the associations of these stress measures with AL are shown to be independent of personality factors, supporting the notion of stress as a significant determinant of AL above and beyond stable characteristics of the individual. Finally, the findings contribute to an ongoing discussion about the consequences of divergence in AL index construction across studies. Specifically, it is suggested that observed stress–AL associations may differ depending on the balance between biomarkers characterized by acute versus chronic stress response.

## CONFLICT OF INTEREST

None declared.

## Data Availability

In accordance with the Act on Processing of Personal Data (Act No. 429 of 31 May 2000) of the Danish Data Protection Agency, data cannot be made publicly available due to considerations for privacy and anonymity of the participants and the sensitive nature of the data. However, an anonymized version of the full data set can be made available upon request to researchers who are qualified to handle confidential information in accordance with the aforementioned Danish Data Protection Agency act. Data are from the CAMB study whose steering group may be contacted at camb@sund.ku.dk.
