# Supplementary material for: Objective and subjective stress, personality, and allostatic load
Source: Brain Behav. 2019 Aug 25;9(9):e01386. doi: 10.1002/brb3.1386 (PMC6749483; doi:10.1002/brb3.1386)
Supplement: Supplementary file 1 [file BRB3-9-e01386-s001.docx]

Supplementary tables S1-S2

| **Table S1.** AL primary mediators modeled by objective and subjective stress, adjusted for personality traits | | | | | | |
| --- | --- | --- | --- | --- | --- | --- |
|  | Model 1 | | Model 2 | | Model 3 | |
|  | β | *p* | β | *p* | β | *p* |
| Objective stress | 0.056 | <.001 | 0.049 | <.001 | 0.052 | <.001 |
| Subjective stress |  |  | 0.032 | .029 | 0.031 | .062 |
| Neuroticism |  |  |  |  | -0.029 | .13 |
| Extraversion |  |  |  |  | 0.010 | .53 |
| Openness |  |  |  |  | -0.038 | .014 |
| Agreeableness |  |  |  |  | -0.001 | .93 |
| Conscientiousness |  |  |  |  | -0.070 | <.001 |
| Sex | 0.042 | .004 | 0.040 | .005 | 0.047 | .002 |
| Age | 0.104 | <.001 | 0.106 | <.001 | 0.107 | <.001 |
| Time of blood draw (AM) | -0.028 | .031 | -0.028 | .032 | -0.031 | .020 |
| Fasting status, fasting | -0.024 | .083 | -0.024 | .082 | -0.024 | .080 |
| Years of education | -0.144 | <.001 | -0.142 | <.001 | -0.124 | <.001 |

| **Table S2.** AL secondary outcomes modeled by objective and subjective stress, adjusted for personality traits. | | | | | | |
| --- | --- | --- | --- | --- | --- | --- |
|  | Model 1 | | Model 2 | | Model 3 | |
|  | β | *p* | β | *p* | β | *p* |
| Objective stress | 0.075 | <.001 | 0.069 | <.001 | 0.072 | <.001 |
| Subjective stress |  |  | 0.029 | .040 | 0.028 | .082 |
| Neuroticism |  |  |  |  | -0.022 | .27 |
| Extraversion |  |  |  |  | 0.016 | .33 |
| Openness |  |  |  |  | -0.039 | .014 |
| Agreeableness |  |  |  |  | -0.007 | .62 |
| Conscientiousness |  |  |  |  | -0.052 | .001 |
| Sex | 0.030 | .034 | 0.029 | .043 | 0.036 | .015 |
| Age | 0.065 | .003 | 0.067 | <.001 | 0.069 | <.001 |
| Time of blood draw (AM) | 0.016 | .23 | 0.016 | .22 | 0.015 | .26 |
| Fasting status, fasting | -0.038 | .005 | -0.038 | .005 | -0.038 | .005 |
| Years of education | -0.186 | <.001 | -0.185 | <.001 | -0.169 | <.001 |
